# Oxytocin-induced modulation of explicit and implicit visual perspective taking

**DOI:** 10.1038/s41598-026-40445-2

**Published:** 2026-02-19

**Authors:** Yulong Huang, Chen Qu, Chunyu Wei, Lara Bardi

**Affiliations:** 1 Center for Studies of Psychological Application, School of Psychology, South China Normal University, Guangzhou, China; 2 Department of Experimental Psychology, Ghent University, Ghent, Belgium; 3 Shenzheng Xinan Middle School (Group) Yanchuan High School, Shenzheng, China

**Keywords:** Neuroscience, Psychology, Psychology

## Abstract

Visual perspective taking (VPT) is a fundamental component of social cognition, allowing individuals to understand environments from diverse viewpoints. Explicit VPT involves deliberately adopting another person’s perspective, whereas implicit VPT reflects the incidental influence of others’ viewpoints when responding from one’s own. Oxytocin (OT), a neuropeptide known for its role in social bonding, has been proposed to influence self–other processing, though its effects on VPT remain unclear. In a double-blind, placebo-controlled study, seventy-nine healthy male participants (Oxytocin = 39, Placebo = 40) completed explicit and implicit VPT tasks. Participants judged object locations from an avatar’s perspective (explicit) or from their own perspective (implicit), in the presence of a human agent or an object. OT administration was associated with reduced accuracy in the explicit task under perspective conflict, reflecting increased egocentric interference. In contrast, in the implicit task, OT was associated with faster and more accurate responses in congruent trials involving a human agent. Together, these findings indicate that oxytocin-related effects on visual perspective taking vary across task demands and social context. Rather than reflecting a general enhancement or impairment of perspective-taking ability, the results provide behavioral evidence consistent with differential modulation of self-other processing.

## Introduction

In everyday life, when we enter the presence of others, we commonly seek to acquire information about them, either consciously or unconsciously. Simultaneously, the presence of others also influences how we perceive and present ourselves, together shaping our perspectives and behavior^1^. This dynamic relies on a fundamental cognitive ability known as visual perspective taking (VPT), a core process of social cognition that enables individuals to present and understand their environment from another person’s viewpoint and take into account what others see (Level 1 perspective taking) and how they see it (Level 2 perspective taking)^2–4^. Successful VPT requires individuals to draw upon both spatial and social information. Spatial information involves knowledge of the relative positions of oneself, others, and the targeted objects within a shared environment^5–8^. Social information, in contrast, shapes the relevance and salience of others’ perspectives depending on contextual and interpersonal factors^9,10^. Importantly, individual differences in VPT abilities have significant practical relevance, for example, in individuals with autism, where difficulties in social communication and perspective taking are hallmark features and are often linked to difficulties in theory of mind (ToM)^11–15^, the ability to attribute beliefs, desires, and mental states to others^16^. However, recent research also argues that the ToM hypothesis alone may not fully explain the social cognitive differences observed in autism^17^. Given the foundational role of VPT in ToM and broader social functioning, identifying potential modulators (e.g., neurobiological factors) that influence perspective-taking is of both theoretical and applied relevance.

One promising neurobiological approach for modulating visual perspective taking is the hypothalamic neuropeptide oxytocin (OT)^18^. OT has garnered significant interest for its role in shaping social and emotional behavior in both animals and humans^19–23^. Widely studied for its involvement in attachment, trust, empathy, and other prosocial behaviors^20,24–27^, OT is often referred to as the “bonding hormone” or “love hormone”. As a key modulator of emotional regulation and social cognition, increasing attention has been directed toward its influence on perspective taking^25,28,29^. While OT’s influence on social behavior is well documented, how it modulates self–other processing and perspective taking remain unclear. Some studies suggest that OT increases attention to others, whereas others report that it enhances or reduces self-related processing. Research focusing on self–other differentiation therefore points to two partially overlapping theoretical accounts, suggesting that OT may either enhance or reduce self-other distinction.

Here we provide a summary of these studies. Several studies have shown that OT enhances other-oriented processing and facilitates the recognition of others’ emotions, empathy, and social understanding. For example, Domes et al. found that OT improved participants’ ability to infer others’ mental states from facial expressions^25,30^. Abu-Akel et al. observed that OT enhanced empathy when participants were explicitly instructed to adopt another’s perspective^28^. Shamay-Tsoory et al. highlighted oxytocin’s role in emotional perspective taking, showing that it increased empathy for out-group adversaries’ pain^31^. Zak et al. found that OT enhances prosocial behavior and moral decision-making, improving altruistic acts toward in-group members^32^. Aydogan et al. reported improved prediction of others’ actions in strategic interactions^33^. Bartz et al. observed that OT increased focus on others at the expense of agentic self-awareness in anxiously attached individuals^34^. These findings collectively highlight oxytocin’s (OT’s) role in enhancing other-related processing. In contrast, fewer studies have examined how OT influences self-oriented processing. Some findings suggest that OT enhances self-related processing: for instance, it has been shown that intranasal OT enhanced positive self-evaluation^35^and increased the differential neural processing of self- vs. celebrity-judgments^36^. However, several studies indicate that OT tends to attenuate self-related biases. Zhao et al. found that OT eliminated self-referential bias in trait judgment tasks, and this effect was also modulated by individual differences in OXTR genotype^37^. Liao et al. showed that OT reduced self-oriented reward learning performance^38^, while also abolishing derogatory judgements of others in object evaluations, but does not affect self-enhancement^39^.

In the specific domain of visual perspective taking, evidence suggests that oxytocin (OT) can modulate self-other distinction, although findings remain mixed. Yue et al. examined the effects of intranasal OT on a visual perspective-taking (VPT) task where participants alternated between self- and other-perspective prompts. They reported that oxytocin reduced self-interference when female participants adopted the perspective of an avatar. However, this effect was not present for male participants^40^. Similarly, Tomova et al. demonstrated that OT improved performance on a perspective-taking task (i.e., the director task) by reducing self-interference when participants adopted the perspective of an avatar in a scene, an effect interpreted as enhanced self-other distinction^41^. In contrast, other studies suggest that OT blurs the boundary between self and other. For instance, Zhao et al. found that OT increases the blurring of self and other during trait perception^42^. De Coster et al. and Ruissen and de Bruijn observed greater merging of self and other in motor simulation tasks under OT administration^43,44^. Conversely, there is also evidence that OT can enhance self-other distinction under certain conditions. In a self-other face differentiation task, Colonnello et al. found that OT lowered the threshold for distinguishing between one’s own and others’ faces (i.e., sharpening self-other distinction)^45^. Further support for this comes from studies showing that OT increased in-group/out-group distinction^46,47^. Taken together, these mixed findings suggest that behavioral effects of oxytocin on perspective-taking may vary with task demands and social context, and highlight the need to better characterize the conditions under which oxytocin modulates perspective-taking.

**Fig. 1:**
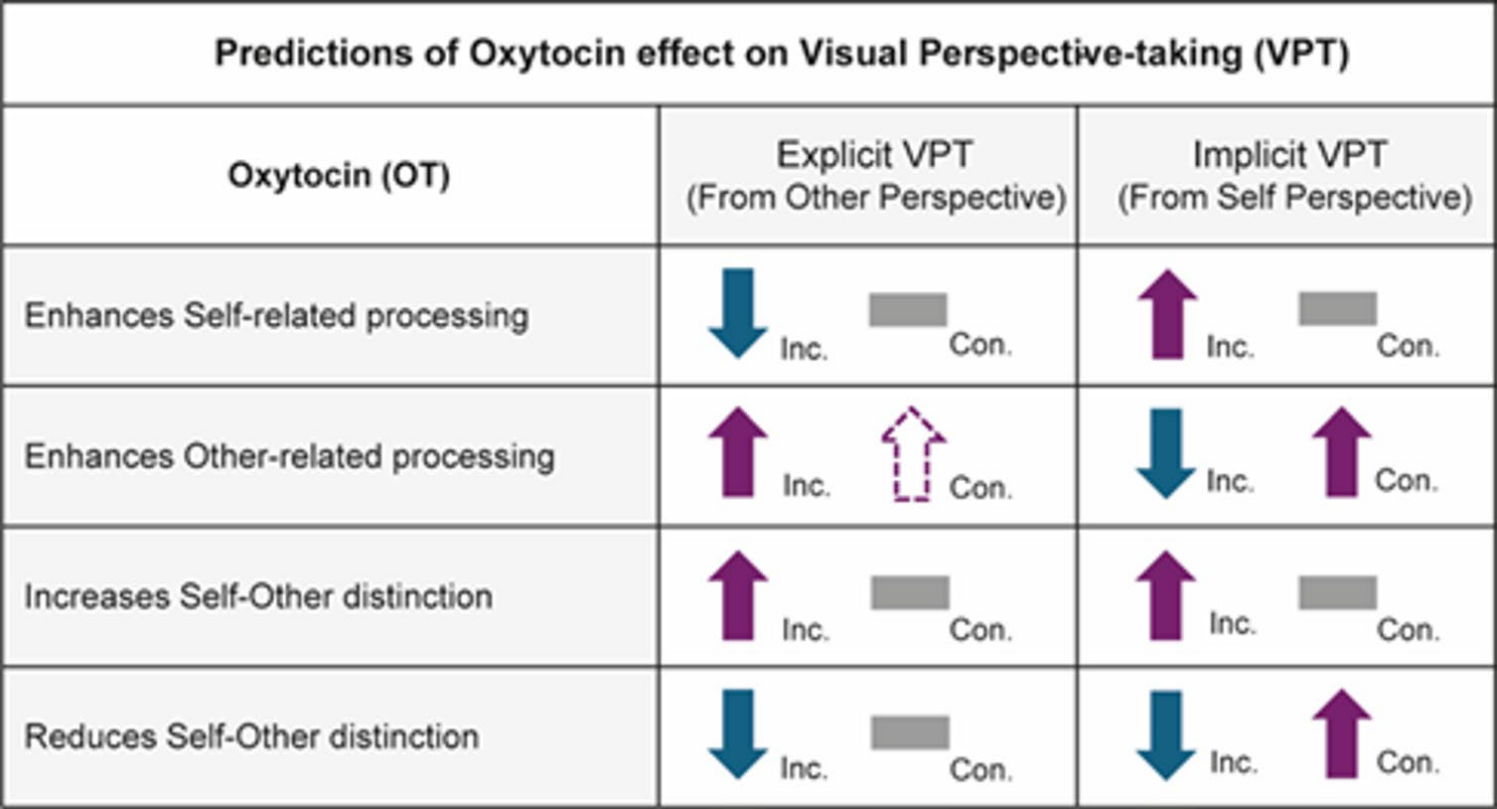
Predicted effects of Oxytocin (OT) on explicit and implicit visuospatial perspective-taking (VPT) tasks under different theoretical accounts of OT-related modulation of self–other processing. The table illustrates predictions for how task performance may change depending on whether OT is associated with enhanced self-related processing, enhanced other-related processing, sharpened self–other distinction, or reduced (blurred) self–other distinction. Purple upward arrows indicate predicted improvements in performance, blue downward arrows indicate predicted reductions in performance, horizontal gray bars indicates predicted minimal effect. Dotted arrows denote effect from null to small. OT effects are expected to be most pronounced in incongruent trials, in which self and other perspectives conflict and demands on self–other differentiation are highest. *Inc.* incongruent trials, *Con.* congruent trials.

The current study investigated the effect of oxytocin (OT) administration on performance in explicit and implicit visuospatial perspective-taking (VPT) tasks. These two forms of VPT differ in their cognitive demands and the relevance of others’ perspectives, providing a behavioral framework for examining how OT may differentially influence self-other related processing. Explicit VPT involves the intentional consideration of another person’s perspective, often requiring individuals to suppress their own viewpoint in favor of adopting another’s. In contrast, implicit VPT can occur spontaneously while participants are required to take their own, egocentric perspective while the perspective of another person may incidentally influence performance, even when it is task-irrelevant. Both tasks involve congruent and incongruent trials, in which perspectives of self and other align or conflict, respectively. Incongruent trials place greater demands on self–other differentiation, as successful performance requires maintaining separation between competing perspectives. Here, we tested whether OT effects vary as a function of task demands and self–other dynamics (see Fig. 1). Specifically, (i) if OT facilitates self-related processing, reduced performance is expected in the explicit task (especially in incongruent trials), where self-perspective must be suppressed. On the other hand, improved performance in the implicit task, which relies on egocentric judgments. (ii) If OT enhances attention to others, performance in the explicit task should improve, particularly in incongruent trials, whereas performance in the implicit task may be reduced in incongruent trials (due to increased interference from the task-irrelevant other perspective), but preserved or modestly improved in congruent trials. (iii) If OT sharpens self-other distinction, performance should improve primarily in incongruent trials across both tasks, where distinguishing between perspectives is critical. Conversely, (iv) if OT reduces or blurs self-other distinction, performance in incongruent trials of both explicit and implicit tasks is expected to decline, reflecting increased interference between self and other perspectives, while congruent trials may be relatively unaffected or even improved in the implicit task.

**Fig. 2:**
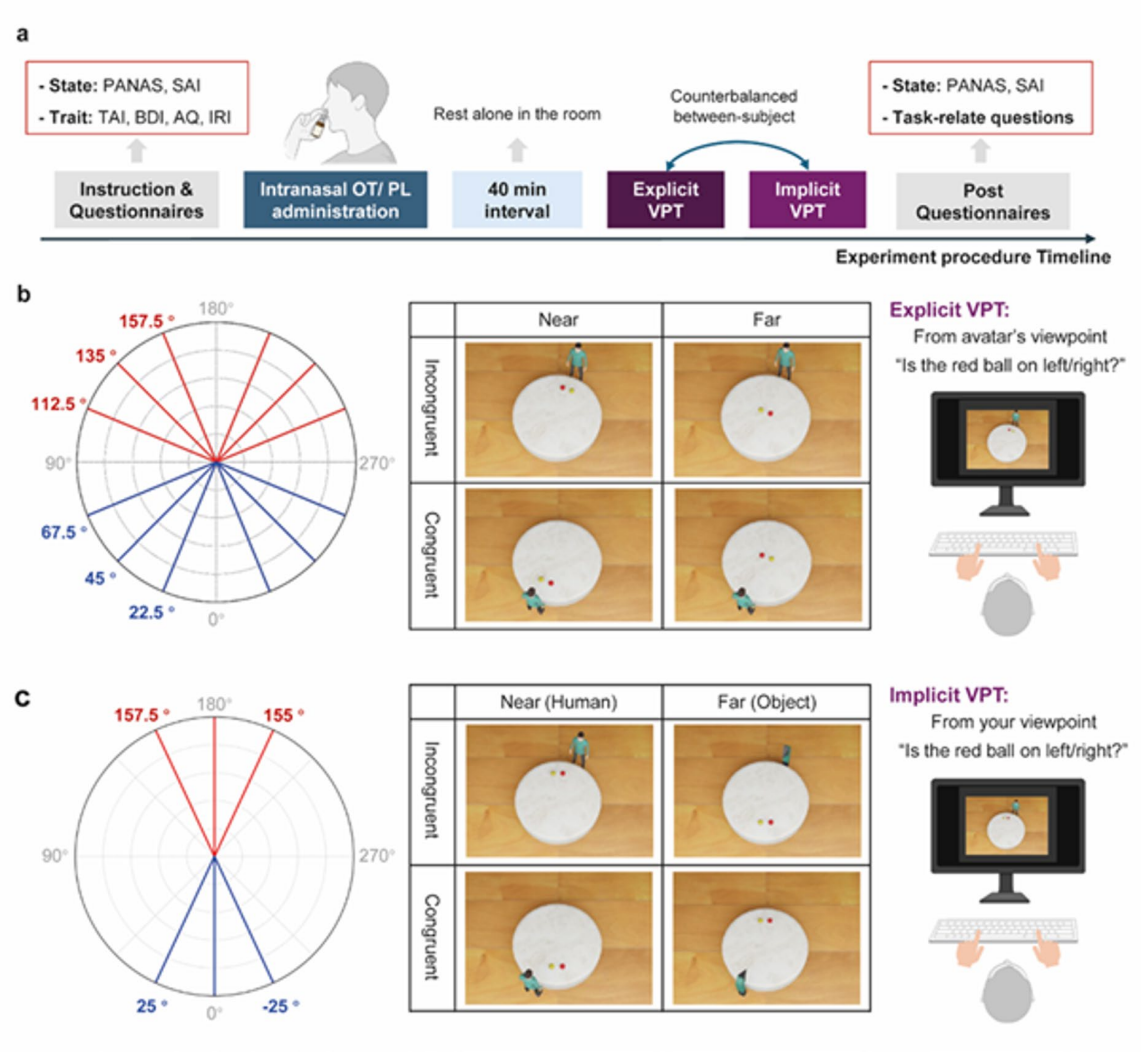
Experimental procedure and task designs. (**a**) Experimental procedure timeline. Participants first completed a series of trait questionnaires, including the Trait Anxiety Inventory (TAI), Beck Depression Inventory (BDI), Autism Spectrum Quotient (AQ), and Interpersonal Reactivity Index (IRI), as well as state-related questionnaires (Positive and Negative Affect Schedule, PANAS, and State Anxiety Inventory, SAI). They were then given instructions on the intranasal spray and self-administered either oxytocin (OT) or placebo (PL). After, participants rested alone in a quiet room for 40 min. Subsequently, they were instructed to perform the explicit and implicit visuospatial perspective-taking (VPT) task, which together lasted approximately 35 min. Lastly, they completed state-related questionnaires again and answered task-related questions. (**b**) Explicit VPT task. The left panel shows the polar plots representing the spatial angles used in the task; blue indicates congruent, and red indicates incongruent trials. The right panel illustrates the task, where participants were instructed to adopt the avatar’s perspective to determine whether the target object (e.g., a red ball) was to the left or right. Example stimuli demonstrate the Congruence (Congruent vs. Incongruent) × Distance (Near vs. Far) conditions. (**c**) Implicit VPT task. The left panel shows the spatial angles used in the task. In the right panel, participants made left/right judgments about the position of the red ball from their own perspective, while a human or object agent remained present in the scene but was irrelevant to the task. In this task, the target was always presented on the participant’s midline, and the manipulations of angular disparity referred only to the position of the human avatar or object. Random interspersed attention-check trials (not illustrated in the example stimuli) required participants to indicate the agent’s position. Example stimuli illustrate the Congruence (Congruent vs. Incongruent) × Distance (Near vs. Far) × Agent (Human vs. Object) conditions.

Using a double-blind, placebo-controlled, within-subject crossover design, we investigated the effects of a single dose of 24 international units (IU) of intranasal oxytocin (IN-OT) on explicit and implicit visuospatial perspective-taking (VPT) abilities. As shown in Fig. 2a, participants first completed a series of trait- and state-related questionnaires to assess potential between-group differences (oxytocin vs. placebo) in emotional state, personality traits, and cognitive flexibility. Participants were then randomly assigned to receive either oxytocin (OT; *n* = 39) or placebo (PL; *n* = 40). Each participant self-administered 24 IU of oxytocin or placebo via intranasal spray under double-blind conditions. The treatment was administered 40 min prior to the VPT tasks, and the task order (explicit vs. implicit) was counterbalanced across participants. Following the tasks, participants filled out post-state-related questionnaires and answered four debriefing questions: (1) Guessing whether they had received OT or PL; (2) Rating the irritation intensity of the intranasal spray from 0 to 10; (3) Rating the influence of the spray’s irritation on task performance from 0 to 10; and (4) whether the tasks order influence their performance (yes or no).

To assess visuospatial perspective-taking, we adapted a task paradigm from previous studies^6,48,49^, which provides a framework for exploring the cognitive processes underlying self-other differentiation. As shown in Fig. 2b, the explicit VPT task required participants to adopt the perspective of an avatar in a scene, whose perspective could be congruent (aligned) or incongruent (conflicting) with the participant’s own. Participants were asked to judge whether a target (i.e., a red ball) was located to the left or to the right from the avatars’ perspective. The task design manipulated both the angular disparity of the avatar (22.5°, 45°, 65.5°, 112.5°, 135°, 157.5°, both clockwise and anticlockwise) and the distance of the target from the avatar (near vs. far). In the implicit VPT task (see Fig. 2c), participants were instructed to make left/right judgment about the position of the red ball from their own perspective, while the avatar’s perspective, which could be congruent or incongruent with that of the participant, remained task-irrelevant. As in the explicit task, the target’s distance and the avatar’s angular position were varied; however, angular differences were simplified to avoid perceptual ambiguity (i.e., the target is clearly on the left or right from the avatar’s perspective). An object condition was included as a control, allowing us to test the social nature of the implicit VPT effect. Additionally, to ensure participants paid attention to the avatar or object, randomly interspersed attention-check trials required participants to indicate the agent’s position. This design enabled us to examine the effect of oxytocin on self- versus other-oriented processing, and how OT modulates performance depending on whether self (Implicit VPT) or other (Explicit VPT) perspectives are prioritized by task demands.

## Results

### Global oxytocin and placebo groups characteristics

As shown in Table 1, independent t-tests revealed no significant differences between the oxytocin (OT) and placebo (PL) groups on demographic variables, baseline personality traits, or pre- and post-treatment measures of mood and anxiety. For most measures, Bayes factors (BF₁₀ < 1) provided evidence in favor of the null hypothesis, supporting the comparability of the two groups, including for measures with p-values approaching the conventional significance threshold (e.g., Interpersonal Reactivity Index, IRI total score). The only exception was the Fantasy subscale of the IRI, which showed a significant group difference (*p* = 0.029, BF_10_ = 1.94), suggesting a baseline difference in imaginative perspective-taking tendencies. Accordingly, Fantasy scores were included as a covariate in subsequent analyses. No significant group differences were found in the self-reported irritation caused by the intranasal spray (OT = 8.28 ± 1.85*SD*, PL = 8.15 ± 1.64*SD*, *t*_(77)_ = 0.336, *p* = 0.738, BF_10_ = 0.245), nor its influence on the task performance (OT = 4.28 ± 2.46*SD*, PL = 3.78 ± 2.55*SD*, *t*_(77)_ = 0.900, *p* = 0.371, BF_10_ = 0.332). The average accuracy of participants guessing whether they had received the placebo or oxytocin was 44.3%, which is below the chance level, confirming the effectiveness of the double-blind procedure. No significant difference was found between groups. Additionally, 78.5% of participants self-reported that the order of explicit and implicit visuospatial perspective-taking (VPT) tasks did not affect their performance. There was no significant difference between groups (OT = 0.85 ± 0.37*SD*, PL = 0.73 ± 0.45*SD*, *t*_(77)_ = 1.308, *p* = 0.195, BF_10_ = 0.294).

**Table 1:** Demographic characteristics and questionnaire scores.

	OT group (*n* = 39)		PL group (*n* = 40)		t-score^1^		*p*-value		BF_10_^2^	
Age (year)	22.00 ± 3.08		21.25 ± 2.60		1.169		0.246		0.422	
Pre-PANAS-positive	31.23 ± 6.44		30.68 ± 7.01		0.367		0.715		0.248	
Pre-PANAS-negative	18.10 ± 6.72		18.68 ± 5.74		− 0.408		0.685		0.251	
Pre-SAI	42.62 ± 5.17		42.33 ± 6.17		0.226		0.821		0.239	
Post-PANAS-positive	30.26 ± 8.07		29.58 ± 7.63		0.386		0.701		0.249	
Post-PANAS-Negative	15.08 ± 5.45		16.25 ± 5.50		− 0.952		0.344		0.346	
Post-SAI	40.72 ± 5.08		41.35 ± 6.79		− 0.467		0.642		0.257	
TAI	43.33 ± 4.85		45.00 ± 6.47		− 1.293		0.200		0.482	
BDI	29.18 ± 4.99		30.38 ± 7.53		− 0.829		0.409		0.315	
AQ	21.95 ± 4.39		22.13 ± 6.22		− 0.145		0.885		0.236	
IRI	58.39 ± 9.21		62.15 ± 9.13		− 1.824		0.072		0.977	
Perspective taking	15.26 ± 3.73		16.40 ± 3.93		− 1.325		0.189		0.499	
Fantasy	15.03 ± 3.41		16.73 ± 3.38		− 2.225		0.029*		1.935	
Empathic concern	16.21 ± 2.64		16.83 ± 2.74		− 1.025		0.309		0.368	
Personal distress	11.90 ± 4.34		12.20 ± 3.51		− 0.341		0.734		0.246	

Data are presented as mean ± standard deviation (SD).

OT, oxytocin; PL, placebo; PANAS, Positive and negative affect schedule; SAI, State Anxiety Inventory; TAI, Trait Anxiety Inventory; BDI, Beck Depression Inventory; AQ, Autism Spectrum Quotient; IRI, Interpersonal Reactivity Index, including subscales: Perspective Taking, Fantasy, Empathic Concern, and Personal Distress.

^1^Frequentist two-tailed Student’s t-test; * indicates *p* < 0.05.

^2^Bayesian two-tailed Student’s t-test; BF₁₀ indicates the Bayes Factor in favor of the alternative hypothesis.

### The effect of oxytocin (OT) on explicit VPT performance

To examine the effect of oxytocin on explicit visuospatial perspective-taking performance, we conducted repeated-measures ANOVAs on both accuracy and reaction time using Frequentist and Bayesian statistical approaches. The model included Treatment (Oxytocin vs. Placebo) as a between-subjects factor, and Distance (Far vs. Near) and Visual congruency (Congruent vs. Incongruent) as within-subjects factors. Given a group difference on the Fantasy subscale of the Interpersonal Reactivity Index (IRI), this variable was included as a covariate to account for individual differences in imaginative perspective-taking (see “Methods” for details on the statistics).

#### OT decreased performance accuracy in the explicit VPT in incongruent trials

**Fig. 3:**
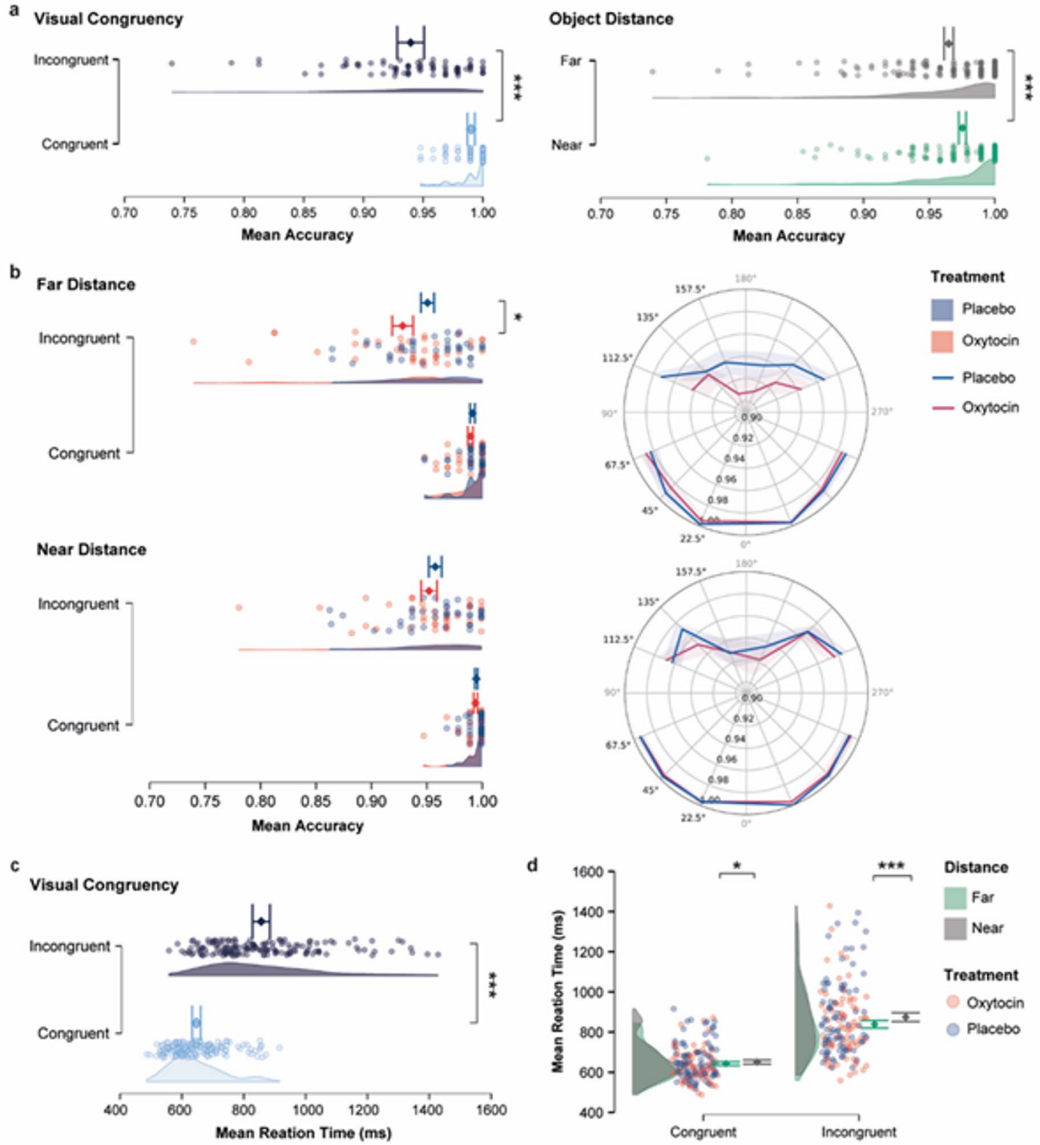
Effects of oxytocin on explicit visuospatial perspective-taking (VPT) performance. (**a**) Mean accuracy (proportion of correct responses) across conditions of visual congruency (Congruent vs. Incongruent) and object distance (Near vs. Far). Participants were significantly less accurate in incongruent compared to congruent trials, and when the target was far compared to near. (**b**) OT administration specifically reduced accuracy in the far-incongruent condition. Polar plots show group differences (Oxytocin vs. Placebo) in accuracy across spatial angles and conditions. (**c**) Reaction time was significantly slower in incongruent compared to congruent trials. (**d**) Reaction time was also slower when the target was near the avatar, with the effect amplified in incongruent trials. Error bars represent standard error of the mean (SEM); diamond markers indicate means. Asterisks indicate significance levels: * *p* < 0.05, ** *p* < 0.005, *** *p* < 0.001. If no asterisk is shown, the effect is not statistically significant.

As shown in Fig. 3a, participants demonstrated a significant main effect of visual congruency on accuracy (F_(1,61)_ = 72.45, *p* < 0.001, *η*_*p*_^2^ = 0.543). They were less accurate on incongruent trials compared to congruent (*M*_congruent_ = 0.993 ± 0.01*SD*, *M*_incongruent_ = 0.948 ± 0.04*SD*). The Bayes Factor (BF_incl_ = 1.462 × 10^+ 14^) provides decisive evidence in favor of the alternative hypothesis, confirming the robustness of the congruency effect. A significant main effect of distance was also found (F_(1,61)_ = 9.31, *p* = 0.003, *η*_*p*_^2^ = 0.132; BF_incl_ = 1209.452), with participants performing more accurately when the target was near the avatar compared to when it was far (*M*_far_ = 0.965 ± 0.03*SD*, *M*_near_ = 0.975 ± 0.02*SD*). However, no significant main effects were observed for treatment (F_(1,61)_ = 72.45, *p* = 0.134, *η*_*p*_^2^ = 0.036, BF_incl_ = 0.939), nor the Fantasy scores included as a covariate (F_(16,61)_ = 0.79, *p* = 0.695, *η*_*p*_^2^ = 0.171, BF_incl_ = 0.331). Interestingly, we observed a significant interaction between treatment and distance (F_(1,61)_ = 5.75, *p* = 0.02, *η*_*p*_^2^ = 0.086; BF_incl_ = 1.428), and a three-way interaction among treatment, distance, and congruency (F_(1,61)_ = 4.57, *p* = 0.036, *η*_*p*_^2^ = 0.070; BF_incl_ = 2.522). While the Bayes Factors offer only anecdotal to moderate evidence for these interactions, these results suggest that the effect of oxytocin on accuracy varies depending on both spatial distance and visual congruency. As shown in Fig. 3b, post-hoc comparisons indicated that this interaction was driven by the incongruent condition. Specifically, when the target was far from the avatar, participants in the oxytocin group were significantly less accurate than those in the placebo group (Incongruent-Far: *M*_OT_ = 0.928 ± 0.06*SD*, *M*_PL_ = 0.951 ± 0.04*SD*, t = − 2.030, *p*_*bonf*_ = 0.047, Cohen’s *d* = − 1.372, 95%CI = [− 2.696, − 0.047]). However, when the target was near, no significant group difference was observed (Incongruent-Near: *M*_OT_ = 0.953 ± 0.05*SD*, *M*_PL_ = 0.958 ± 0.04*SD*, t = − 0.515, *p*_*bonf*_ = 0.608, Cohen’s *d* = − 0.272, 95% CI = [− 1.307, 0.763]). No significant differences were found in congruent trials, regardless of distance, suggesting that OT decreased performance accuracy when participants had to take the avatar’s incongruent perspective, and when the target was spatially far from the avatar.

#### OT did not modulate reaction time in the explicit VPT task

As shown in Fig. 3c, there was a significant main effect of visual congruency (F_(1,61)_ = 163.20, *p* < 0.001, *η*_*p*_^2^ = 0.728; BF_incl_ = 8.936 × 10^+ 13^), with participants responding more slowly in incongruent trials than in congruent trials (*M*_congruent_ = 646.61 ± 86.45*SD*, *M*_incongruent_ = 856.33 ± 177.35*SD*). A significant main effect of distance was also found (F_(1,61)_ = 21.49, *p* < 0.001, *η*_*p*_^2^ = 0.261, BF_incl_ = 3.716 × 10^+ 9^), showing that responses were slower when the target was far from the avatar compared to when it was near (*M*_far_ = 758.98 ± 134.10*SD*, *M*_near_ = 738.19 ± 117.25*SD*). Covariate Fantasy scores also significantly predicted reaction time (F(16, 61) = 2.25, *p* = 0.012, *η*_*p*_^2^ = 0.371; BF_incl_ = 1.163), indicating a reliable association between Fantasy and response speed. No significant main effect of treatment was observed (F_(1,61)_ = 0.61, *p* = 0.437, *η*_*p*_^2^ = 0.010, BF_incl_ = 0.499), indicating that oxytocin did not affect overall reaction time. However, a significant interaction between distance and congruency was found (F_(1,61)_ = 5.80, *p* = 0.019, *η*_*p*_^2^ = 0.087, BF_incl_ = 6383.706). Specifically, post-hoc comparisons (see Fig. 3d) revealed that the distance effect was significant in both congruent trials (*M*_far_ = 650.67 ± 91.76*SD*, *M*_near_ = 642.56 ± 82.79*SD*, t = 2.863, *p*_*bonf*_ = 0.034, Cohen’s *d* = 0.404, 95%CI = [0.128, 0.680]) and incongruent trials (*M*_far_ = 874.25 ± 192.95*SD*, *M*_near_ = 838.59 ± 163.64*SD*, t = 3.918, *p*_*bonf*_ < 0.001, Cohen’s *d* = 1.186, 95%CI = [0.592, 1.780]). However, the effect was more pronounced in incongruent trials, where participants had to overcome the avatar’s conflicting perspective. No significant interaction was found for treatment × congruency (F_(1,61)_ = 0.03, *p* = 0.873, *η*_*p*_^2^ < 0.001, BF_incl_ = 0.735), treatment × distance (F_(1,61)_ = 1.34, *p* = 0.251, *η*_*p*_^2^ = 0.022, BF_incl_ = 1.039), or the three-way interaction (F_(1,61)_ = 1.12, *p* = 0.295, *η*_*p*_^2^ = 0.018, BF_incl_ = 0.133). These results suggest that, although the effects of spatial distance and visual congruency were robust in the Explicit VPT task, oxytocin did not modulate reaction times when participants were required to adopt the perspective of others.

Taken together, findings in the explicit VPT task indicate that oxytocin administration reduced accuracy when participants judge targets that were far from an incongruent human avatar. This effect was not observed in congruent trials and did not extend to reaction times. These results can be interpreted in two ways (see Fig. 1). First, OT may increase the salience of the self-perspective, leading to stronger egocentric interference and greater difficulty inhibiting one’s own viewpoint when adopting the avatar’s perspective. Alternatively, the findings are consistent with increased overlap or blurring between self- and other-related representations, which may impair the maintenance of a clearly distinct other-oriented perspective under conditions of high perspective conflict.

### The effect of oxytocin on implicit VPT performance

In the implicit VPT task, participants were required to respond to the same question (“Is the target to the right or to the left?”) but from their own perspective. Here, we aim to examine whether the presence of a task-irrelevant avatar implicitly influences performance and whether intranasal oxytocin administration modulates this effect. As with the explicit task, we conducted repeated-measures ANOVAs on both accuracy and reaction times using Frequentist and Bayesian statistical approaches. The model included Treatment (Oxytocin vs. Placebo) as a between-subjects factor, and Agent (Human vs. Object), Distance (Far vs. Near), and Visual congruency (Congruent vs. Incongruent) as within-subjects factors. The fantasy subscale was included as a covariate to account for individual differences in imaginative perspective-taking (see “Methods” for details on the statistics). To ensure participants remained attentive during the task, we analyzed performance on attention-check trials, in which they were intermittently asked to identify the location of an agent (human or object) within the scene. These trials served as a control measure of general task engagement.

#### Oxytocin reduced the accuracy to identify agent location in general

Participants demonstrated strong overall performance in the attention-check trials, with a high mean accuracy of 94.9% ± 4.6SD, indicating consistent task engagement across conditions and groups. Analysis of accuracy revealed a significant main effect of agent type (F(1,76) = 4.357, *p* = 0.040, *η*_*p*_^2^ = 0.054; BF_incl_ = 0.749), with slightly lower accuracy when the agent was a human avatar compared to an object (*M*_human_ = 0.944 ± 0.06*SD*, *M*_object_ = 0.955 ± 0.04*SD*). A significant main effect of treatment was also found (F(1,76) = 13.043, *p* < 0.001, *η*_*p*_^2^ = 0.146; BF_incl_ = 23.449), where participants in the oxytocin group showed lower accuracy than those in the placebo group (*M*_OT_ = 0.933 ± 0.06*SD*, *M*_PL_ = 0.966 ± 0.03*SD*). While this suggests a general attentional effect of oxytocin, it is important to note that overall performance remained high, and these trials were unrelated to perspective-taking demands. No significant effects of covariate Fantasy scores were found (F(1,76) = 1.935, *p* = 0.168, *η*_*p*_^2^ = 0.025; BF_incl_ = 0.419), nor was there a significant interaction between treatment and agent type (F(1,76) = 1.031, *p* = 0.313, *η*_*p*_^2^ = 0.013; BF_incl_ = 0.569). In addition to accuracy, we analyzed reaction times to further characterize general attentional effects. Result revealed a significant main effect of agent type (F(1,76) = 5.156, *p* = 0.026, *η*_*p*_^2^ = 0.064; BF_incl_ = 1.188), with slower responses to the human avatar than to the object (*M*_human_ = 1311.42 ± 197.65*SD*, *M*_object_ = 1288.46 ± 210.80*SD*). There was a significant interaction between agent type and fantasy (F(1,76) = 4.127, *p* = 0.046, *η*_*p*_^2^ = 0.052; BF_incl_ = 1.143), suggesting that imaginative traits modestly modulate the effect of agent on response speed. Overall, these findings confirm that participants were attentive and engaged throughout the task. The differences in accuracy and reaction time based on agent type may reflect the increased social salience of human avatars. While oxytocin was associated with a small reduction in accuracy, this occurred in the low-level control trials and the overall performance remained high.

#### OT increased accuracy in congruent trials when a human avatar was present

**Fig. 4:**
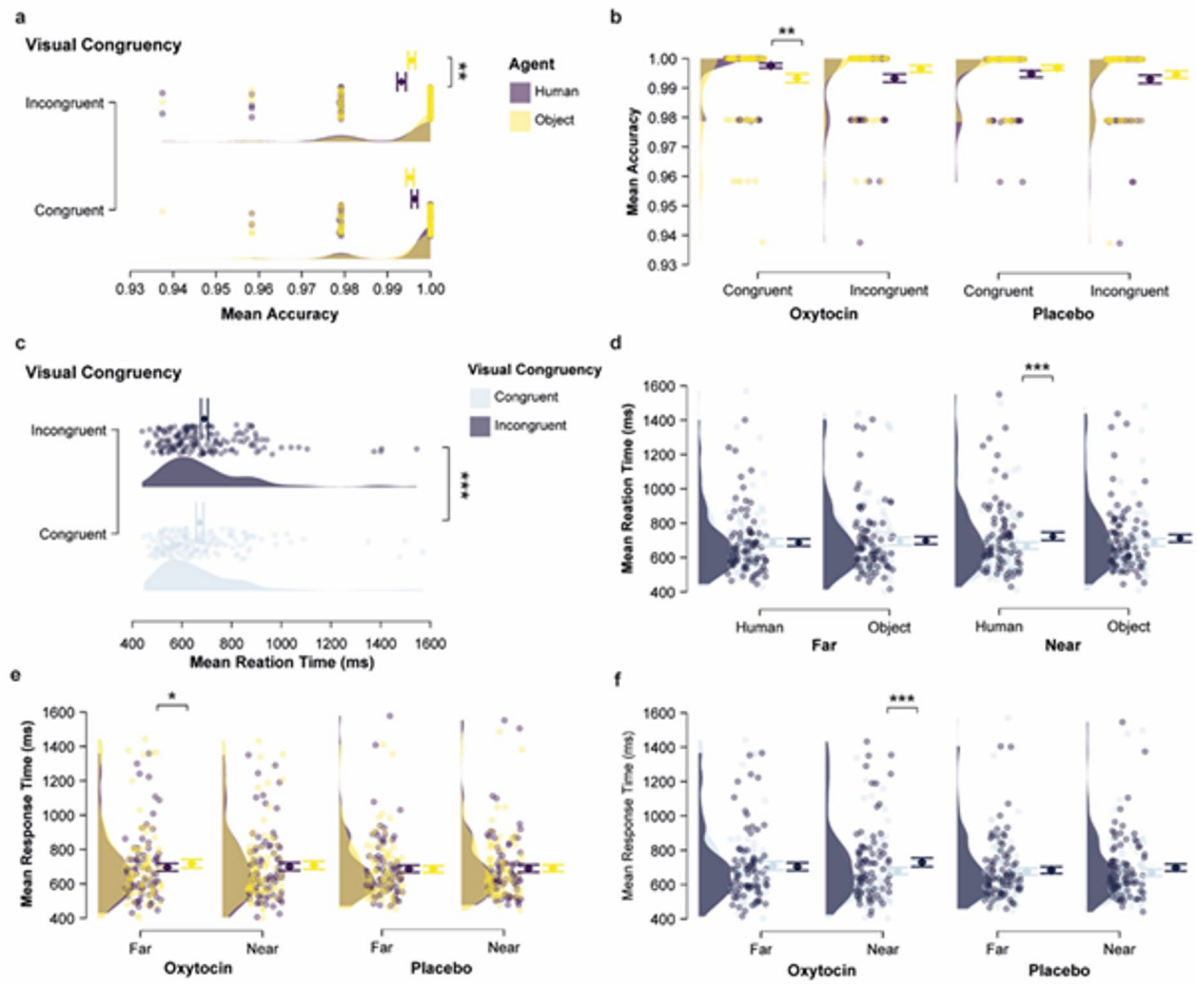
Effects of oxytocin on implicit visuospatial perspective-taking (VPT) performance. (**a**) Mean accuracy (proportion of correct response) showing the interaction between visual congruency (Congruent vs. Incongruent) and agent type (Human vs. Object). Participants were significantly less accurate in incongruent trials when a human avatar was present. (**b**) OT administration increased mean accuracy in congruent trials when a human avatar was present. (**c**) Reaction times were significantly slower in incongruent compared to congruent trials. (**d**) The congruency effect on reaction time was only significant when the target was near the human avatar. (**e**) OT administration sped up responses when the target was far from a human avatar compared to an object. (**e**) The congruency effect on reaction time was only significant within the OT group when the target was near the agent, regardless of whether it was a human or object. Error bars represent standard error of the mean (SEM); diamond markers indicate means. Asterisks indicate significance levels: * *p* < 0.05, ** *p* < 0.005, *** *p* < 0.001. If no asterisk is shown, the effect is not statistically significant.

In the implicit VPT task, where participants responded from their own perspective, analysis of accuracy revealed no significant main effects of treatment (F(1,61) = 0.13, *p* = 0.725, *η*_*p*_^2^ = 0.02; BF_incl_ = 0.027), agent type (F(1,61) = 0.00, *p* = 0.972, *η*_*p*_^2^ < 0.001; BF_incl_ = 0.035), distance (F(1,61) = 1.27, *p* = 0.263, *η*_*p*_^2^ = 0.020; BF_incl_ = 0.131), or congruency (F(1,61) = 0.07, *p* = 0.795, *η*_*p*_^2^ = 0.001; BF_incl_ = 0.059). The Bayes Factors (< 0.3) for these tests provide moderate to strong evidence in favor of the null hypothesis for these main effects. However, as shown in Fig. 4a, a significant interaction between agent type and congruency was observed (F(1,61) = 8.56, *p* = 0.005, *η*_*p*_^2^ = 0.123; BF_incl_ = 0.071). While the Frequentist result was statistically robust, the Bayes Factor provides moderate evidence against the alternative hypothesis, suggesting this interaction should be interpreted with caution. Post-hoc comparisons showed that the congruency effect was not significant for either agent type after correction (*p*_*bonf*_ > 0.064), therefore, follow-up analyses focused on agent-type differences within congruency conditions. Participants were less accurate in incongruent trials involving a human avatar compared to an object (*t* = − 2.079, *p*_*bonf*_ = 0.042, Cohen’s *d* = − 0.336, 95% CI [− 0.556, − 0.116]). No significant difference was found in congruent trials (*t* = 1.875, *p*_*bonf*_ = 0.066, Cohen’s *d* = 0.336, 95% CI [− 0.103, 0.775]). Furthermore, a significant three-way interaction between treatment, agent type, and congruency was observed (F(1,61) = 6.44, *p* = 0.014, *η*_*p*_^2^ = 0.096; BF_incl_ = 0.035). Again, the Bayesian analysis provided only weak support for this effect. Nevertheless, in post-hoc comparisons within the oxytocin group, accuracy in congruent trials was significantly higher when it was a human avatar compared to an object (Congruent: *M*_human_ = 0.998 ± 0.004*SD*, *M*_object_ = 0.993 ± 0.012*SD*; *t* = 3.253, *p*_*bonf*_ = 0.002, Cohen’s *d* = 0.672, 95% CI = [0.233, 1.111], see Fig. 4b). This effect was not observed in the placebo group. Despite the low Bayes Factors suggesting weak Bayesian support, the observed pattern is consistent with a potential oxytocin-related facilitation of implicit performance in socially congruent contexts, particularly when a human agent is present.

#### OT speeds up responses when a human avatar was present

As shown in Fig. 4c, analysis of reaction time revealed a significant main effect of congruency (F(1,61) = 7.99, *p* = 0.006, *η*_*p*_^2^ = 0.116; BF_incl_ = 275128.898), with slower responses in incongruent trials than in congruent trials (*M*_congruent_ = 682.68 ± 188.73*SD*, *M*_incongruent_ = 703.30 ± 194.32*SD*). The Bayes Factor provided decisive support for this congruency effect. A marginal main effect of agent type was also observed (F(1,61) = 3.69, *p* = 0.060, *η*_*p*_^2^ = 0.057; BF_incl_ = 1.168), with participants tending to respond faster when a human avatar was present compared to an object (*M*_human_ = 689.66 ± 189.82*SD*, *M*_object_ = 696.39 ± 194.51*SD*), though this result was supported only by anecdotal Bayesian evidence. No significant main effect was found for treatment (F(1,61) = 0.73, *p* = 0.396, *η*_*p*_^2^ = 0.012; BF_incl_ = 0.003) or distance (F(1,61) = 0.09, *p* = 0.763, *η*_*p*_^2^ = 0.002; BF_incl_ = 129.961). However, two interaction effects were significant: Distance x Congruency (F(1,61) = 4.68, *p* = 0.034, *η*_*p*_^2^ = 0.071; BF_incl_ = 912.954) and Agent type x Distance x Congruency (F(1,61) = 5.04, *p* = 0.028, *η*_*p*_^2^ = 0.076; BF_incl_ = 29.225). Post-hoc comparisons (see Fig. 4d) showed that the congruency effect was significant only when the target was near a human avatar (Near: *M*_congruent_ = 664.16 ± 178.73*SD*, *M*_incongruent_ = 719.04 ± 211.82*SD*; *t* = − 3.526, *p*_*bonf*_ = 0.001, Cohen’s *d* = − 1.190, 95% CI = [− 1.852, − 0.528]).

Next, we examined the effects of oxytocin on task performance. Although the overall four-way interaction was not significant, two significant three-way interactions involving treatment effect were found: Treatment × Distance × Congruency (F(1,61) = 5.21, *p* = 0.026, *η*_*p*_^2^ = 0.079; BF_incl_ = 0.081) and Treatment × Distance × Agent (F(1,61) = 4.82, *p* = 0.032, *η*_*p*_^2^ = 0.073; BF_incl_ = 0.002). Because the corresponding Bayes factors provided moderate to strong evidence in favor of the null hypothesis, the significant frequentist effects motivated cautious, exploratory follow-up analyses. To further characterize the Treatment × Distance × Agent interaction, we first examined the agent-type effect. As shown in Fig. 4e, post-hoc comparisons revealed that oxytocin significantly sped up responses when a human avatar was present, but only when the target was far (Far: *M*_human_ = 696.12 ± 195.42*SD*, *M*_object_ = 717.36 ± 223.76*SD*; *t* = -2.810, *p*_*bonf*_ = 0.007, Cohen’s *d* = − 1.028, 95%CI = [− 1.745, − 0.311]). This effect was not present in the near condition (Near: *M*_human_ = 700.44 ± 195.35*SD*, *M*_object_ = 708.23 ± 208.32*SD*; *t* = − 1.341, *p*_*bonf*_ = 0.630, Cohen’s *d* = − 0.187, 95% CI = [− 0.944, 0.570]), nor in the placebo group. This suggests that oxytocin-related effects on response speed may depend on the presence of a socially relevant agent at greater spatial distances. In contrast, post-hoc comparison of the Treatment × Distance × Congruency interaction indicated that oxytocin increased the congruency effect (i.e., faster responses in congruent than incongruent trials), only when the target was near the agent, regardless of whether it was human or object (see Fig. 4f, Near: *M*_congruent_ = 679.42 ± 182.59*SD*, *M*_incongruent_ = 729.33 ± 219.54*SD*; *t* = -3.686, *p*_*bonf*_ < 0.001, Cohen’s *d* = − 0.818, 95% CI [− 1.253, − 0.383]). No congruency effect was found when the target was far (Far: *M*_congruent_ = 709.16 ± 215.31*SD*, *M*_incongruent_ = 704.14 ± 204.67*SD*; *t* = 0.382, *p*_*bonf*_ = 0.704, Cohen’s *d* = 0.079, 95% CI [− 0.329, 0.487]), nor in the placebo group. Taken together, although these effects were not strongly supported by Bayesian evidence and should therefore be interpreted with caution, the observed patterns are consistent with oxytocin-related modulation that might depend on both social relevance and context.

In summary, these findings suggest that the effect of oxytocin on implicit perspective-taking performance is context-dependent. Specifically, accuracy improvements were observed in congruent trials involving a human avatar; faster responses occurred in the presence of distant human agents, suggesting increased social salience under oxytocin. Although Bayesian analyses often favored the null hypothesis, the results of frequentist analyses suggest subtle oxytocin-related modulation of self–other processing, even when participants responded from their own perspective. These results can be interpreted in two non-mutually exclusive ways (see again Fig. 1). First, oxytocin may increase interference from other-related cues, making it harder for participants to suppress the influence of the avatar’s perspective, particularly in cognitively demanding situations (i.e., leading to a stronger congruency effect when the target was near the agents). Second, oxytocin may enhance the integration or blurring of self-other representation, which can benefit performance in socially aligned contexts (i.e., higher accuracy in congruent trials with the human avatar and faster responses when the target was far from the human avatar).

## Discussion

Oxytocin (OT) has been proposed to influence how individuals process self- and other-related information, yet its effects on visual perspective-taking remain debated. Prior work has questioned whether oxytocin sharpens distinctions between self and other or instead promotes greater overlap between perspectives. To address these issue, the current study examined whether a single dose (24IU) of intranasal OT modulates behavioral performance on explicit and implicit visual perspective-taking (VPT) tasks. In the explicit task, participants were required to intentionally adopt the perspective of another human agent; in the implicit task, they responded from their own perspective, while the presence of another agent (human or object) could implicitly interfere with their performance. By employing both VPT tasks, we were able to examine whether OT administration is associated with distinct patterns of behavior that have been linked to self–other processing in prior work (see Fig. 1).

Overall, our findings reveal a context-dependent effect of OT on perspective-taking. In the explicit task, OT administration reduced accuracy when participants had to judge a far-positioned target from an incongruent human avatar’s perspective (see Fig. 3b). This accuracy drop occurred without changes in reaction time, suggesting that OT did not slow participants generally, but instead made it harder to suppress the self-perspective when adopting another’s viewpoint. In contrast, in the implicit task, OT improved performance by increasing accuracy in congruent trials involving a human avatar (see Fig. 4b) and sped up responses when the target was far from the human avatar (see Fig. 4e). Additionally, OT amplified the congruency effect on reaction times, but only when the target was spatially near the agent, irrespective of whether it was an human avatar or an object, suggesting increased sensitivity to alignment between self and other perspectives.

Taken together, these findings are in line with the hypothesis that OT downregulates or reduces self-other distinction (see Fig. 1). However, the fact that OT affects incongruent trials in the explicit task and congruent trials in the implicit task suggests that OT-related effects may be context dependent. In other words, its effect on self–other processing depends not only on the presence of perspective conflict, but also on whether the task requires separation or alignment of self and other representations. In the explicit task, successful performance requires active suppression of the self-perspective and the maintenance of a clear self–other distinction. Incongruent trials therefore place maximal demands on this separation, which could explain why OT-related effects (blurring self-other distinction) emerged primarily under conditions of perspective conflict. In contrast, in the implicit task the other’s perspective was task-irrelevant and does not need to be actively suppressed. Under these conditions, blurring of self-other distinction may have less detrimental consequences during incongruent trials. This pattern highlights that OT does not exert a uniform effect on perspective taking, but instead modulates performance as a function of how self–other overlap interacts with task demands.

Such dual effects are consistent with models suggesting that OT enhances automatic interpersonal alignment^50^and social salience^51^, but can hinder executive control and perspective-shifting when cognitive demands are high^19,20,52^. The observed improvements in implicit task performance involving human avatars are compatible with prior evidence that OT enhances social attention^53^, empathy^26,31^, mind-reading^25^and sensitivity to socially relevant cues. At the same time, the reduced accuracy observed in the explicit task under high conflict underscores that OT does not uniformly enhance social cognition. When tasks require inhibiting one’s own perspective and maintaining a clear distinction between self and other, OT administration was associated with behavioral patterns consistent with reduced self–other differentiation. An additional consideration is the well-documented role of oxytocin in modulating in-group bias^46,47^. Although the avatar in the present study were not explicitly defined as in-group or out-group members, it is possible that participants implicitly treated human avatars (compared to objects) as socially relevant or group-consistent agents. Given that the effects in the implicit VPT task were not consistently supported by Bayesian evidence, these findings should be interpreted with caution.

Recent work has questioned whether implicit VPT paradigms truly reflect spontaneous mentalizing. Using a mirror-reflection dot perspective task that equates visual access between participants and avatars, Wang et al. demonstrated that altercentric interference persists even when visual perspective differences are eliminated, supporting a submentalizing account based on directional attentional orienting rather than implicit theory of mind^54^. Aware of the ongoing debate on implicit perspective taking and the submentalizing account, we included a control condition in which the avatar was replaced by an object. We found that the congruency effect on reaction times was larger in the social (avatar) condition than in the non-social (object) condition. Oxytocin further modulated the congruency effect irrespective of agent type, however, this modulation was more pronounced in the avatar condition. Although these results certainly do not resolve the debate, they do not support the submentalizing hypothesis. Recent work further suggests that interoceptive sensitivity plays an important role in resolving conflicts between self and other. Gardner et al. (2024) showed that individuals with lower interoceptive sensitivity exhibited greater errors during perspective-taking when self and other viewpoints conflicted, an effect attributed to increased self–other blurring^55^. At the same time, oxytocin (OT) has been suggested to modulate interoceptive awareness^56,57^. Although interoceptive processes were not directly assessed in the present study, this convergence of findings points to a potential link between bodily self-representation and the ability to resolve self–other conflict, and suggests a possible mechanism through which OT might exert its effects.

Our findings diverge from those of Yue et al.^40^, who reported OT reduced self–other interference during explicit perspective-taking in female participants, but not in males. In our study, which included only male participants, OT increased self-interference in incongruent trials, leading to reduced accuracy. This discrepancy highlights the importance of considering sex as a moderating variable in OT research. Indeed, previous work has well-documented sex-dependent effects of OT on social cognition, including judgment^58,59^, social motivation^60,61^, cooperation and competition^62,63^, and relationship maintenance^64^, which can differ markedly between males and females^65^. These differences may reflect the modulatory influence of gonadal hormones or sex-specific variations in OT receptor distribution^66^. Beyond the effect of OT, our findings from the implicit task are noteworthy. Prior work has suggested that implicit effects of another agent’s perspective diminish or disappear when participants are repeatedly instructed to take their own perspective (e.g., in blocked designs)^67^, however, we observed clear interference from a task-irrelevant human agent. This suggests that implicit social influence in VPT may be more robust than previously assumed. However, this effect may have been reinforced by the inclusion of attention-check trials, although infrequent (8 of 48), may have increased the attentional salience of the avatar and encouraged spontaneous consideration of its perspective.

Several limitations should be noted. As in many OT studies, we administered a standard single dose (24 IU), limiting our ability to assess dosage-dependent effects or long-term effects. The exclusively males sample restricts generalizability, and replication in female and mixed-sex samples is essential. In addition, the implicit VPT task involved multiple spatial and agent-related manipulations that may have introduced cognitive complexity, potentially overloading participants’ attentional resources. Moreover, some observed effects, particularly higher-order interactions, were modest in size and not always supported by Bayesian analyses, requiring cautious interpretation and future replication of the effects. Finally, individual traits such as fantasy appeared to modulate performance, indicating that interindividual differences may shape OT-related behavioral effects. Importantly, the present study does not aim to identify underlying neural or biological mechanisms. Rather, it provides a systematic behavioral characterization of how oxytocin-related effects on perspective-taking vary across task demands and social contexts. Future studies combining behavioral paradigms with neurophysiological measures, direct manipulations of social affiliation, and assessments of interoceptive processing may help to further constrain theoretical models of oxytocin’s role in self–other processing. Given the relevance to populations with impaired self–other processing, such as autism spectrum disorder, applying these paradigms in clinical contexts may help inform targeted, neurobiologically informed interventions.

## Conclusion

In conclusion, our findings suggest that oxytocin administration is associated with context-dependent differences in perspective-taking performance, consistent with the idea that OT blurs self and other representations. Specifically, OT-related effects varied as a function of task demands. In the explicit task, OT was associated with increased interference from the self-perspective when participants were required to adopt another’s viewpoint, particularly under conditions of high cognitive demand (i.e., incongruent trials). In contrast, in the implicit task, OT-related effects emerged primarily in socially congruent contexts, where self and other perspectives were aligned. Together, these findings indicate that oxytocin modulates visuospatial perspective-taking in a manner that depends on both task demands and the degree of conflict between self and other perspectives. By directly contrasting explicit and implicit perspective-taking within a controlled behavioral paradigm, the present study helps delineate the conditions under which oxytocin may be associated with either facilitation or impairment of perspective-taking. Although the observed effects were modest and not consistently supported by Bayesian evidence, identifying the task contexts in which oxytocin-related behavioral differences emerge may help inform future mechanistic and clinical research on self–other processing.

## Methods

### Participants

Participants were recruited via online advertisement platforms within South China Normal University. All participants filled out a pre-screening form. The exclusion criteria included neurologic and psychiatric symptoms, color blindness or psychotropic medication, and the use of any substantial medication or other substance (including heavy smoking). Participants were instructed to refrain from smoking or drinking (except water) for at least 2 h before the experiment. The sample size was determined based on a priori power analysis conducted in G*Power v.3.1^68^ for a three-way mixed analysis of variance (ANOVA) with the following parameters: effect size = 0.25; significance level = 0.05; power = 0.95; Number of Groups = 2; Number of Measurements = 4. The analysis indicated a required sample size of thirty-six participants per group. Eighty male participants were recruited. One participant was excluded from the final analysis dataset due to technical issues that prevented proper data recording. Thus, the final dataset included 79 male participants aged 18–34 years (mean age = 21.62, SD = 2.85). Only male participants were included to avoid potential gender-dependent differences in response to oxytocin administration^69,70^. All participants provided written informed consent and received monetary compensation for their participation. Ethical approval for the study was approved by Ethical Committee of South China Normal University (Reference ID: SCNU-PSY-2022-346), and all experiments were performed in accordance with relevant guidelines and regulations.

### Experimental procedure

We employed a randomized, double-blind, placebo-controlled, between-subjects design to investigate the effects of a single dose of intranasal oxytocin (IN-OT) on explicit and implicit visuospatial perspective-taking (VPT) abilities (within-subjects). To account for potential between-group differences (Oxytocin vs. Placebo) in emotional state, personality traits, and cognitive flexibility, participants completed validated Chinese versions of the following questionnaires before treatment: the Trait Anxiety Inventory (TAI)^71^, the Beck Depression Inventory (BDI)^72^, the Autism Spectrum Quotient (AQ)^73^, and the Interpersonal Reactivity Index (IRI)^74,75^. The IRI was specifically included to examine potential correlations between task performance and trait empathy. To further control for any confounding effects of emotional state changes, participants also completed the Positive and Negative Affect Schedule (PANAS)^76^and the State Anxiety Inventory (SAI)^71^at two time points: before treatment and after completing the tasks. Participants were randomly assigned to either the oxytocin (OT) group (*n* = 39) or the placebo (PL) group (*n* = 40). They were instructed to self-administer 24 international units (IU) of oxytocin or a placebo (double-blinded). The treatment was administered 40 min before the start of the explicit and implicit VPT tasks, with the task order counterbalanced between participants. During the 40-minute waiting period, participants rested alone in the experimental room to minimize social or emotional stimulation (see Fig. 2a). At the end of the experiment, participants were asked to answer several task-related questions, including: (1) Guessing whether they had received the placebo or oxytocin; (2) Rating the irritation intensity of the intranasal spray on a scale from 0 (not at all) to 10 (extremely); (3) Rating the influence of the spray’s irritation on task performance on a scale from 0 (not at all) to 10 (extremely); and (4) whether the explicit and implicit VPT tasks order influence their performance.

### Treatment administration

A 24IU dose of oxytocin (OT acetate spray, supplied by Sichuan Defeng Pharmaceutical Co., Ltd, China) was administered dissolved in sodium chloride and glycerol. After participants fully understood the instructions of the nasal spray given by the experimenter, the intranasal spray was self-administered as six 0.1 ml puffs (three puffs per nostril) with 30-s intervals between puffs. The 24IU dose of oxytocin and the administration procedure have followed the recommendations in the literature^23,77^. The placebo spray, identical in appearance, contained the same composition as the oxytocin spray except for the absence of the neuropeptide (also supplied by Sichuan Defeng Pharmaceutical Co., Ltd, China). The study was conducted in a double-blind manner. Spray bottles for both conditions were identical and coded by an individual not involved in the experiment. The coding was revealed only after the experiment was completed.

### Explicit visuospatial perspective-taking (VPT) task

We adapted a spatial perspective-taking task with a design similar to that used in Kessler and Thompson^6^, Kessler and Rutherford^78^, and Wang et al.^49^. Visual stimuli were created using Blender, a free and open-source 3D graphics software (https://www.blender.org/). Each image depicted an avatar positioned near a round table on a wooden background, with the avatar standing at 12 different angular positions (22.5°, 45°, 65.5°, 112.5°, 135°, 157.5°, clockwise and anticlockwise). Two balls—a red ball and a yellow ball—were placed in front of the avatar, with the red ball serving as the target for participants’ responses. From the avatar’s perspective, the red ball appeared on either the left or the right. The distances of the two balls were also manipulated to be either near or far from the avatar, resulting in 48 unique images (12 angles × 2 red ball positions × 2 distances) used as stimuli.

During the experiment, participants were seated approximately 70 cm from the screen and were instructed not to move their heads during the task. After receiving instructions, they were first given two example images (e.g., the avatar at 0° with the balls either near or far) and conducted 12 practice trials that included all experimental angles (see Fig. 2b). The practice trials were identical to the formal task, but feedback was provided in each trial to ensure participants understood the task.

In the explicit VPT task, participants were instructed to take the perspective of the avatar in the images and determine whether the target (i.e., the red ball) is located to the left (pressing the “f” key) or right (pressing the “j” key) from the avatars’ point of view. Each trial began with a 700-ms fixation cross, followed by the presentation of the stimulus. Participants were instructed to respond as quickly and accurately as possible, although no time limit was imposed. The formal task consisted of 8 blocks of 48 trials each (384 total trials), presented in random order within each block. No feedback was provided in each trial; however, at the end of each block, participants received feedback on their average accuracy for that block. They were instructed to maintain an average accuracy of at least 90% throughout the task. Participants were allowed self-paced breaks between blocks.

### Implicit visuospatial perspective-taking (VPT) task

The implicit VPT task followed a similar overall structure to the explicit task but employed a reduced set of angular variations (25°, 0°, − 25°), which were congruent or incongruent with the participant’s position. As shown in Fig. 2c, stimuli featured either a human avatar or an object as the agent, and the distance of the target from the agent (near vs. far) was counterbalanced. Importantly, in the implicit task the target (red ball) was always presented at 0° relative to the participant, such that it was aligned with the participant’s egocentric midline. The manipulation of angular disparity therefore refers exclusively to the spatial position of the avatar or object, and not to the position of the target. This design resulted in 48 unique stimuli (6 angles × 2 red ball positions × 2 distances × 2 agents).

Prior to the formal task, participants were shown example images (i.e., an avatar or object at 0° with balls near) and completed 12 practice trials (6 per agent condition) to familiarize themselves with the task. During the task, participants were seated centrally in front of the screen and instructed to respond from their own perspective, indicating whether the red ball was positioned to the left or right side by pressing the “f” (left) or “j” (right) key. Each trial began with a 700-ms fixation cross followed by the stimulus. Participants completed 16 blocks (8 for human avatars and 8 for objects) of 24 trials each (384 total trials), presented in random order within the block. The block order was counterbalanced between participants. Feedback on average accuracy was provided at the end of each block, and participants were allowed self-paced breaks.

To ensure participants paid attention to the agent’s presence, attention-check trials (*n* = 8 per block; 128 trials in total, human = 64, object = 64) were included and randomly appeared within the block, requiring participants to identify the agent’s position in the current trial. Moreover, the reduced set of angular positions relative to the explicit task was chosen to minimize perceptual ambiguity, ensuring that the target was clearly positioned to the left or right from the avatar’s perspective. The inclusion of the object condition served as a non-social control, allowing us to assess the social specificity of implicit VPT effects.

### Data cleaning and statistical analysis

Data cleaning and preprocessing were conducted in R (version 4.4.1). Raw behavioral data were prepared according to the dependent variables. For the analysis of accuracy, trials with reaction time (RT) below 200 ms or above 3000 ms were excluded, as these were considered invalid responses. This led to the exclusion of 109 trials (0.45%) in the explicit VPT task and 136 trials (0.36%) in the implicit VPT task across all participants. For the analysis of reaction time, only correct-response trials within the 200–3000 ms window were retained, ensuring that RT measures reflected valid and accurate performance. In total, 1009 trials (3.34%) were excluded from the explicit VPT task and 284 trials (0.94%) from the implicit VPT task. The final dataset included only valid trials meeting these criteria and was then used for all subsequent statistical analysis.

#### Frequentist statistics

Behavioral data were analyzed using both Frequentist and Bayesian statistical approaches. For Frequentist analysis, we conducted repeated-measures ANCOVAs using the aov_car() function from the *afex* package^79^in R (Type III sums of squares). For the explicit VPT task, the model included Treatment (Oxytocin vs. Placebo) as a between-subjects factor, and Distance (Near vs. Far) and Congruency (Congruent vs. Incongruent) as within-subjects factors. For the implicit VPT task, the model included Treatment (Oxytocin vs. Placebo) as a between-subjects factor, and Agent type (Human vs. Object), Distance, and Congruency as within-subjects factors. In both models, the Fantasy subscale from the Interpersonal Reactivity Index (IRI) was included as a mean-centered covariate to account for individual differences in imaginative perspective-taking. Subject ID was specified as a random effect to appropriately model within-subject dependencies. Significant effects were followed up with Bonferroni-adjusted post hoc tests using the *emmeans* package^80^. Pairwise contrasts were calculated for all relevant main effects and interactions, with results reported as means, standard deviation, t-ratios, *p*-values, *Cohen’s d*, and their 95% confidence intervals. Effect sizes were interpreted based on conventional benchmarks (small: d = 0.2; medium: d = 0.5; large: d ≥ 0.8). Significance was set at *p* < 0.05, unless otherwise stated.

#### Bayesian statistics

For Bayesian analysis, we conducted it in JASP (version 0.19.3)^81^ using the same model structures as the frequentist analysis. We computed Bayes Factors (BF_10_) to quantify the relative evidence for the alternative hypothesis (H_1_) over the null (H_0_), where BF_10_ = p(data|H_1_)/p(data|H_0_)^82^. A BF_10_ > 1 indicates support for H₁, while a BF_10_ < 1 favors H_0_. Values between 1/3 and 3 are interpreted as anecdotal; values > 3 or < 1/3 reflect moderate to strong evidence in favor of H_1_ or H_0_, respectively. For example, BF_10_ = 10 means the data are 10 times more likely under H_1_ than under H_0_. For repeated-measures ANOVA, inclusion Bayes Factors (BF_incl_) were reported to evaluate the added predictive value of each model term (main effects and interactions) by comparing models with and without the factor^83^. Default priors in JASP were applied based on the test type. Bayesian results are presented alongside Frequentist outcomes in the Results section, providing a complementary perspective on the strength and direction of the observed effects.

## Data Availability

The behavioral data that support the findings of this study is available on OSF: https://osf.io/k3t2f/.

## Code Availability

All codes used to generate the analyses is available on OSF: https://osf.io/k3t2f/.

## References

[1] Kampis, D. & Southgate, V. Altercentric cognition: how others influence our cognitive processing. *Trends Cogn. Sci.* **24**, 945–959 (2020). 32981846 10.1016/j.tics.2020.09.003

[2] Masangkay, Z. S. et al. The early development of inferences about the visual percepts of others. *Child Dev.* **45**, 357–366 (1974). 4837714

[3] Flavell, J. H. The development of knowledge about visual perception. In *Nebraska Symposium on Motivation. Nebraska Symposium on Motivation*, vol. 25, 43–76 (1977). 753993

[4] Flavell, J. H., Everett, B. A., Croft, K. & Flavell, E. R. Young children’s knowledge about visual perception: further evidence for the level 1-Level 2 distinction. *Dev. Psychol.* **17**, 99–103 (1981).

[5] Zacks, J. M., Vettel, J. M. & Michelon, P. Imagined viewer and object rotations dissociated with event-related fMRI. *J. Cogn. Neurosci.* **15**, 1002–1018 (2003). 14614811 10.1162/089892903770007399

[6] Kessler, K. & Thomson, L. A. The embodied nature of spatial perspective taking: embodied transformation versus sensorimotor interference. *Cognition* **114**, 72–88 (2010). 19782971 10.1016/j.cognition.2009.08.015

[7] Kessler, K., Wang, H. & and Spatial perspective taking is an embodied Process, but not for everyone in the same way: differences predicted by sex and social skills score. *Spat. Cogn. Comput.* **12**, 133–158 (2012).

[8] Job, X. E., Kirsch, L. P. & Auvray, M. Spatial perspective-taking: insights from sensory impairments. *Exp. Brain Res.* **240**, 27–37 (2022). 34716457 10.1007/s00221-021-06221-6 PMC8803716

[9] Cavallo, A., Ansuini, C., Capozzi, F., Tversky, B. & Becchio, C. When Far becomes near: perspective taking induces social remapping of Spatial relations. *Psychol. Sci.* **28**, 69–79 (2017). 27864372 10.1177/0956797616672464

[10] Woo, B. M., Tan, E., Yuen, F. L. & Hamlin, J. K. Socially evaluative contexts facilitate mentalizing. *Trends Cogn. Sci.* **27**, 17–29 (2023). 36357300 10.1016/j.tics.2022.10.003

[11] Baron-Cohen, S., Campbell, R., Karmiloff‐Smith, A., Grant, J. & Walker, J. Are children with autism blind to the mentalistic significance of the eyes? *Br. J. Dev. Psychol*. **13**, 379–398 (1995).

[12] Frith, C. D. & Frith, U. Social cognition in humans. *Curr. Biol.* **17**, R724–R732 (2007). 17714666 10.1016/j.cub.2007.05.068

[13] Senju, A. Spontaneous theory of mind and its absence in autism spectrum disorders. *Neuroscientist* **18**, 108–113 (2012). 21609942 10.1177/1073858410397208 PMC3796729

[14] Frith, C. D. & Frith, U. Mapping mentalising in the brain. In *The Neural Basis of Mentalizing* (eds Gilead, M. & Ochsner, K. N.) 17–45. 10.1007/978-3-030-51890-5_2 (Springer International Publishing, 2021).

[15] Gao, S., Wang, X. & Su, Y. Examining whether adults with autism spectrum disorder encounter multiple problems in theory of mind: a study based on meta-analysis. *Psychon. Bull. Rev.* **30**, (2023). 10.3758/s13423-023-02280-8 37101097

[16] Premack, D. & Woodruff, G. Does the chimpanzee have a theory of mind? *Behav. Brain Sci.* **1**, 515–526 (1978).

[17] Long, E. L., Catmur, C. & Bird, G. The theory of mind hypothesis of autism: A critical evaluation of the status quo. *Psychol. Rev.* (2025). 10.1037/rev0000532 39786849

[18] Sartorius, A. M. et al. An evolutionary timeline of the oxytocin signaling pathway. *Commun. Biol.* **7**, 1–13 (2024). 38632466 10.1038/s42003-024-06094-9 PMC11024182

[19] Bartz, J. A., Zaki, J., Bolger, N. & Ochsner, K. N. Social effects of oxytocin in humans: context and person matter. *Trends Cogn. Sci.* **15** (7), 301–309 (2011). 21696997 10.1016/j.tics.2011.05.002

[20] Ma, Y., Shamay-Tsoory, S., Han, S. & Zink, C. F. Oxytocin and social adaptation: insights from neuroimaging studies of healthy and clinical populations. *Trends Cogn. Sci.* **20**, 133–145 (2016). 26616296 10.1016/j.tics.2015.10.009

[21] Leppanen, J., Ng, K. W., Tchanturia, K. & Treasure, J. Meta-analysis of the effects of intranasal Oxytocin on interpretation and expression of emotions. *Neurosci. Biobehav. Rev*. **78**, 125–144 (2017). 28467893 10.1016/j.neubiorev.2017.04.010

[22] Kang, H., Sartorius, A. M., Deilhaug, E., Walle, K. M. & Quintana, D. S. A multiverse meta-analysis of intranasal oxytocin administration studies. 10.31219/osf.io/89fgw (2024). 10.1016/j.biopsycho.2025.109112 40848964

[23] Quintana, D. S. et al. Advances in the field of intranasal oxytocin research: lessons learned and future directions for clinical research. *Mol. Psychiatry*. **26**, 80–91 (2021). 32807845 10.1038/s41380-020-00864-7 PMC7815514

[24] Kosfeld, M., Heinrichs, M., Zak, P. J., Fischbacher, U. & Fehr, E. Oxytocin increases trust in humans. *Nature* **435** (7042), 673–676 (2005). 15931222 10.1038/nature03701

[25] Domes, G., Heinrichs, M., Michel, A., Berger, C. & Herpertz, S. C. Oxytocin improves mind-reading in humans. *Biol. Psychiatry*. **61**, 731–733 (2007). 17137561 10.1016/j.biopsych.2006.07.015

[26] Hurlemann, R. et al. Oxytocin enhances amygdala-dependent, socially reinforced learning and emotional empathy in humans. *J. Neurosci.* **30**, 4999–5007 (2010). 20371820 10.1523/JNEUROSCI.5538-09.2010 PMC6632777

[27] Zik, J. B. & Roberts, D. L. The many faces of oxytocin: implications for psychiatry. *Psychiatry Res.* **226**, 31–37 (2015). 25619431 10.1016/j.psychres.2014.11.048

[28] Abu-Akel, A., Palgi, S., Klein, E., Decety, J. & Shamay-Tsoory, S. Oxytocin increases empathy to pain when adopting the other- but not the self-perspective. *Soc. Neurosci.* **10**, 7–15 (2015). 25103924 10.1080/17470919.2014.948637

[29] Geng, Y. et al. Oxytocin facilitates empathic- and self-embarrassment ratings by attenuating amygdala and anterior Insula responses. *Front. Endocrinol.* **9**, 572 (2018). 10.3389/fendo.2018.00572 PMC6190868 30356869

[30] Domes, G. et al. Oxytocin attenuates amygdala responses to emotional faces regardless of valence. *Biol. Psychiatry*. **62**, 1187–1190 (2007). 17617382 10.1016/j.biopsych.2007.03.025

[31] Shamay-Tsoory, S. G. et al. Giving peace a chance: Oxytocin increases empathy to pain in the context of the Israeli–Palestinian conflict. *Psychoneuroendocrinology* **38**, 3139–3144 (2013). 24099859 10.1016/j.psyneuen.2013.09.015

[32] Zak, P. J., Stanton, A. A. & Ahmadi, S. Oxytocin increases generosity in humans. *PLoS One*. **2**, e1128 (2007). 17987115 10.1371/journal.pone.0001128 PMC2040517

[33] Aydogan, G. et al. Oxytocin promotes altruistic punishment. *Soc. Cognit. Affect. Neurosci.* **12**, 1740–1747 (2017). 28981891 10.1093/scan/nsx101 PMC5714236

[34] Bartz, J. A. et al. Differential effects of oxytocin on agency and communion for anxiously and avoidantly attached individuals. *Psychol. Sci.* **26**, 1177–1186 (2015). 26122122 10.1177/0956797615580279

[35] Cardoso, C., Ellenbogen, M. A. & Linnen, A. M. Acute intranasal oxytocin improves positive self-perceptions of personality. *Psychopharmacology* **220**, 741–749 (2012). 22012170 10.1007/s00213-011-2527-6

[36] Liu, Y., Sheng, F., Woodcock, K. A. & Han, S. Oxytocin effects on neural correlates of self-referential processing. *Biol. Psychol.* **94**, 380–387 (2013). 23965321 10.1016/j.biopsycho.2013.08.003

[37] Zhao, W. et al. Oxytocin modulation of self-referential processing is partly replicable and sensitive to oxytocin receptor genotype. *Prog. Neuropsychopharmacol. Biol. Psychiatry*. **96**, 109734 (2020). 31415827 10.1016/j.pnpbp.2019.109734

[38] Liao, Z., Huang, L. & Luo, S. Intranasal oxytocin decreases self-oriented learning. *Psychopharmacology* **238**, 461–474 (2021). 33156402 10.1007/s00213-020-05694-7

[39] Wu, Y., van Dijk, E. & Zhou, X. Evaluating self- vs. other-owned objects: the modulatory role of Oxytocin. *Biol. Psychol.* **92**, 179–184 (2013). 23182874 10.1016/j.biopsycho.2012.11.011

[40] Yue, T., Jiang, Y., Yue, C. & Huang, X. Differential effects of Oxytocin on visual perspective taking for men and women. *Front. Behav. Neurosci.* **11**, 228 (2017). 29187816 10.3389/fnbeh.2017.00228 PMC5694773

[41] Tomova, L., Heinrichs, M. & Lamm, C. The other and me: effects of Oxytocin on self-other distinction. *Int. J. Psychophysiol.* **136**, 49–53 (2019). 29550334 10.1016/j.ijpsycho.2018.03.008

[42] Zhao, W. et al. Oxytocin blurs the self-other distinction during trait judgments and reduces medial prefrontal cortex responses. *Hum. Brain. Mapp.* **37**, 2512–2527 (2016). 27016006 10.1002/hbm.23190 PMC6867482

[43] De Coster, L., Mueller, S. C., T’Sjoen, G., De Saedeleer, L. & Brass, M. The influence of Oxytocin on automatic motor simulation. *Psychoneuroendocrinology* **50**, 220–226 (2014). 25240207 10.1016/j.psyneuen.2014.08.021

[44] Ruissen, M. I. & de Bruijn, E. R. A. Is it me or is it you? Behavioral and electrophysiological effects of Oxytocin administration on self-other integration during joint task performance. *Cortex* **70**, 146–154 (2015). 26026705 10.1016/j.cortex.2015.04.017

[45] Colonnello, V., Chen, F. S., Panksepp, J. & Heinrichs, M. Oxytocin sharpens self-other perceptual boundary. *Psychoneuroendocrinology* **38**, 2996–3002 (2013). 24064220 10.1016/j.psyneuen.2013.08.010

[46] De Dreu, C. K. W. et al. The neuropeptide Oxytocin regulates parochial altruism in intergroup conflict among humans. *Science* **328**, 1408–1411 (2010). 20538951 10.1126/science.1189047

[47] Ten Velden, F. S., Daughters, K. & De Dreu, C. K. W. Oxytocin promotes intuitive rather than deliberated Cooperation with the in-group. *Horm. Behav.* **92**, 164–171 (2017). 27288835 10.1016/j.yhbeh.2016.06.005

[48] Kessler, K. & Rutherford, H. The two forms of visuo-spatial perspective taking are differently embodied and subserve different Spatial prepositions. *Front. Psychol.* **1**, 213 (2010). 21833268 10.3389/fpsyg.2010.00213 PMC3153818

[49] Wang, H., Callaghan, E., Gooding-Williams, G., McAllister, C. & Kessler, K. Rhythm makes the world go round: an MEG-TMS study on the role of right TPJ theta oscillations in embodied perspective taking. *Cortex* **75**, 68–81 (2016). 26722994 10.1016/j.cortex.2015.11.011

[50] Spengler, F. B. et al. Oxytocin facilitates reciprocity in social communication. *Soc. Cognit. Affect. Neurosci.* **12**, 1325–1333 (2017). 28444316 10.1093/scan/nsx061 PMC5597889

[51] Shamay-Tsoory, S. G. & Abu-Akel, A. The social salience hypothesis of Oxytocin. *Biol. Psychiatry*. **79** (3), 194–202 (2016). 26321019 10.1016/j.biopsych.2015.07.020

[52] Olff, M. et al. The role of Oxytocin in social bonding, stress regulation and mental health: an update on the moderating effects of context and interindividual differences. *Psychoneuroendocrinology* **38**, 1883–1894 (2013). 23856187 10.1016/j.psyneuen.2013.06.019

[53] Yao, S. et al. Oxytocin modulates attention switching between interoceptive signals and external social cues. *Neuropsychopharmacol* **43**, 294–301 (2018). 10.1038/npp.2017.189 PMC5729568 28836577

[54] Wang, W., Shangguan, C., Sun, Z., Yang, K. & Zhou, B. Attentional orienting rather than spontaneous perspective taking: A mirror-reflection dot perspective task reveals submentalizing. *Psychol. Res.* **90**, 6 (2025). 41359248 10.1007/s00426-025-02219-9

[55] Gardner, M., Mostafa, I. & Corcoran, J. Heartfelt mentalising: Interoception modulates the ability to resolve conflict between self- and other- perspectives. 10.31234/osf.io/dgbqf (2024).

[56] Betka, S. et al. Impact of intranasal oxytocin on interoceptive accuracy in alcohol users: an attentional mechanism? *Social Cogn. Affect. Neurosci.* **13**(4), 440–448 (2018). 10.1093/scan/nsy027 PMC5928407 29618101

[57] Zhou, M. et al. Intranasal Oxytocin improves interoceptive accuracy and Heartbeat-Evoked potentials during a cardiac interoceptive task. *Biol. Psychiatry Cogn. Neurosci. Neuroimaging*. **9**, 1019–1027 (2024). 38839034 10.1016/j.bpsc.2024.05.004

[58] Hoge, E. A. et al. Gender moderates the effect of Oxytocin on social judgments. *Hum. Psychopharmacol. Clin. Exp*. **29** (3), 299–304 (2014). 10.1002/hup.2402 24911580

[59] Gao, S. et al. Oxytocin,the peptide that bonds the sexes also divides them. *Proc. Natl. Acad. Sci.* **113** (27), 7650–7654 (2016). 27325780 10.1073/pnas.1602620113 PMC4941426

[60] Theodoridou, A., Rowe, A. C. & Mohr, C. Men perform comparably to women in a perspective taking task after administration of intranasal Oxytocin but not after placebo. *Front Hum. Neurosci.* **7**, (2013). 10.3389/fnhum.2013.00197 PMC3664327 23754995

[61] Preckel, K., Scheele, D., Kendrick, K. M., Maier, W. & Hurlemann, R. Oxytocin facilitates social approach behavior in women. *Front. Behav. Neurosci.* **8**, (2014). 10.3389/fnbeh.2014.00191 PMC4034412 24904342

[62] Fischer-Shofty, M., Levkovitz, Y. & Shamay-Tsoory, S. G. Oxytocin facilitates accurate perception of competition in men and kinship in women. *Soc. Cognit. Affect. Neurosci.* **8**, 313–317 (2013). 22446301 10.1093/scan/nsr100 PMC3594723

[63] Scheele, D. et al. Opposing effects of Oxytocin on moral judgment in males and females. *Hum. Brain. Mapp.* **35**, 6067–6076 (2014). 25094043 10.1002/hbm.22605 PMC6868938

[64] Yao, S. et al. Oxytocin makes females, but not males, less forgiving following betrayal of trust. *Int. J. Neuropsychopharmacol.* **17**, 1785–1792 (2014). 24916520 10.1017/S146114571400090X

[65] Caldwell, H. K. Oxytocin and sex differences in behavior. *Curr. Opin. Behav. Sci.* **23**, 13–20 (2018).

[66] Macdonald, K. S. & Sex Receptors, and attachment: A review of individual factors influencing response to Oxytocin. *Front. Neurosci.* **6**, (2013). 10.3389/fnins.2012.00194 PMC3541513 23335876

[67] Samson, D., Apperly, I. A., Braithwaite, J. J., Andrews, B. J. & Bodley Scott, S. E. Seeing it their way: evidence for rapid and involuntary computation of what other people see. *J. Exp. Psychol. Hum. Percept. Perform.* **36**, 1255–1266 (2010). 20731512 10.1037/a0018729

[68] Faul, F., Erdfelder, E., Buchner, A. & Lang, A. G. Statistical power analyses using G*Power 3.1: tests for correlation and regression analyses. *Behav. Res. Methods*. **41**, 1149–1160 (2009). 19897823 10.3758/BRM.41.4.1149

[69] Ditzen, B. et al. Sex-specific effects of intranasal Oxytocin on autonomic nervous system and emotional responses to couple conflict. *Soc. Cognit. Affect. Neurosci.* **8**, 897–902 (2013). 22842905 10.1093/scan/nss083 PMC3831552

[70] MacDonald, K. S. Sex, receptors, and attachment: a review of individual factors influencing response to Oxytocin. *Front. NeuroSci.* **6**, 194 (2013). 23335876 10.3389/fnins.2012.00194 PMC3541513

[71] Speilberger, C. D., Gorsuch, R. L., Lushene, R., Vagg, P. R. & Jacobs, G. A. Manual for the state-trait anxiety inventory. *Palo Alto CA Consult. Psychol.* (1983).

[72] Beck, A. T., Steer, R. A., Ball, R. & Ranieri, W. F. Comparison of Beck depression Inventories-IA and-II in psychiatric outpatients. *J. Pers. Assess.* **67**, 588–597 (1996). 8991972 10.1207/s15327752jpa6703_13

[73] Baron-Cohen, S., Wheelwright, S., Skinner, M., Martin, J. & Clubley, E. The Autism-Spectrum quotient (QA): evidence from asperger syndrome/high functioning autism, males and females, scientists and mathematicians’: errata. *J. Autism Dev. Disord.* https://psycnet.apa.org/record/2002-12228-011 (2001). 10.1023/a:1005653411471 11439754

[74] Davis, M. H. Interpersonal Reactivity Index (1980).

[75] Siu, A. M. H. & Shek, D. T. L. Validation of the interpersonal reactivity index in a Chinese context. *Res. Social Work Pract.* **15**, 118–126 (2005).

[76] Watson, D., Clark, L. A. & Carey, G. Positive and negative affectivity and their relation to anxiety and depressive disorders. *J. Abnorm. Psychol.* **97**, 346 (1988). 3192830 10.1037//0021-843x.97.3.346

[77] Guastella, A. J. et al. Recommendations for the standardisation of Oxytocin nasal administration and guidelines for its reporting in human research. *Psychoneuroendocrinology* **38**, 612–625 (2013). 23265311 10.1016/j.psyneuen.2012.11.019

[78] Kessler, K. The two forms of visuo-spatial perspective taking are differently embodied and subserve different Spatial prepositions. *Front. Psychol.* **1**, (2010). 10.3389/fpsyg.2010.00213 PMC3153818 21833268

[79] Henrik, S., Ben, B., Jake, W., Frederik, A. & Ben-Shachar M. S. afex: Analysis of Factorial Experiments. (2012).

[80] Lenth, R. emmeans Estimated Marginal Means, aka Least-Squares Means. *R package version 1.11.2* (2025).

[81] Love, J. et al. Graphical statistical software for common statistical designs. *J. Stat. Softw.* **88**, 1–17 (2019).

[82] Kruschke, J. K. Bayesian analysis reporting guidelines. *Nat. Hum. Behav.* **5**, 1282–1291 (2021). 34400814 10.1038/s41562-021-01177-7 PMC8526359

[83] Rouder, J. N., Morey, R. D., Speckman, P. L. & Province, J. M. Default Bayes factors for ANOVA designs. *J. Math. Psychol.* **56**, 356–374 (2012).

